# Relevance of GDF15 as a biomarker for clinical outcomes after bariatric surgery

**DOI:** 10.1530/JOE-25-0010

**Published:** 2025-07-01

**Authors:** Paula Urones, Paula Juiz-Valiña, Elena Outeiriño-Blanco, María Jesús García-Brao, Vanesa Balboa-Barreiro, Fernando Cordido, Susana Sangiao-Alvarellos

**Affiliations:** ^1^ Universidade da Coruña (UDC), Grupo de Fisiopatoloxía Endócrina, Nutricional e Médica, CICA-Centro Interdisciplinar de Química e Bioloxía, A Coruña, Spain; ^2^ Universidade da Coruña (UDC), Grupo de Fisiopatoloxía Endócrina, Nutricional e Médica, Departamento de Fisioterapia, Medicina e Ciencias Biomédicas, Facultade de Fisioterapia, A Coruña, Spain; ^3^ Grupo de Enfermedades Endocrinas, Nutricionales y Metabólicas. Instituto de Investigación Biomédica de A Coruña (INIBIC), As Xubias, A Coruña, Spain; ^4^ Complexo Hospitalario Universitario A Coruña (CHUAC), Departamento de Endocrinoloxía, A Coruña, Spain; ^5^ Complexo Hospitalario Universitario A Coruña (CHUAC), Departamento de Cirurxía Xeral, Hospital Universitario A Coruña, A Coruña, Spain; ^6^ Unidade de Apoio á Investigación, Grupo de Investigación en Enfermería e Coidados na Saúde, Grupo de Investigación en Reumatoloxía e Saúde (GIR-S) Complexo Hospitalario Universitario de A Coruña (CHUAC) e Universidade da Coruña (UDC), A Coruña, Spain

**Keywords:** bariatric surgery, GDF15, diabetes, hypertension

## Abstract

Bariatric surgery (BS) is the most effective long-term approach for weight loss and improvement of obesity-related comorbidities. Growth differentiation factor 15 (GDF15), a cytokine that enhances insulin sensitivity and reduces food intake, is a promising therapeutic target for obesity. This study assessed changes in circulating GDF15 levels in obesity and following BS, examining their associations with anthropometric, clinical, and biochemical parameters. Circulating GDF15 levels were measured in normal-weight individuals and patients with obesity before BS and at 3, 6 and 12 months post-surgery. Correlation analyses and linear mixed models were used to investigate variations in circulating GDF15 levels and to identify variables associated with GDF15 concentration. Circulating GDF15 levels were elevated in patients with obesity compared to normal-weight individuals and were higher in men than in women. In the combined cohort of obese and normal-weight individuals, circulating GDF15 levels positively correlated with weight, BMI, fat mass, glucose markers, C-reactive protein, transaminases, triglycerides, urea, creatinine and uric acid, and negatively correlated with apolipoprotein A and total, HDL and LDL cholesterol. In the obese cohort, however, circulating GDF15 levels showed a negative correlation with fat mass, while other associations persisted. After BS, circulating GDF15 levels significantly decreased, particularly in patients with hypertension or type 2 diabetes (T2D). One year post-surgery, ΔGDF15 was negatively associated with BMI and positively with excess weight loss and excess BMI loss. In conclusion, BS significantly reduces circulating GDF15 levels, particularly in patients with hypertension or T2D, indicating an association with clinical improvement after BS.

## Introduction

Obesity is characterized by an excessive accumulation of fat within adipose tissue, which is associated with low-grade chronic inflammation and acts as a risk factor for various diseases. The main obesity-related complications include T2D, metabolic syndrome (MetS), hypertension (HBP), dyslipidaemia, metabolic dysfunction-associated steatotic liver disease (MASLD), certain types of cancer, obstructive sleep apnoea (OSA) and osteoarthritis (Apovian 2016, Cercato & Fonseca 2019).

Growth differentiation factor-15 (GDF15) is a stress-response cytokine and a member of the transforming growth factor beta superfamily that is expressed in multiple tissue including liver, intestine, white (WAT) and brown (BAT) adipose tissue, kidneys, and placenta (Wang *et al.* 2021). Circulating GDF15 levels are elevated in individuals with obesity and are even higher in those with obesity and T2D, which may reflect a compensatory anti-inflammatory mechanism during early stages of metabolic disease (Carstensen *et al.* 2010). Treatment or overexpression of GDF15 exerts protective effects against obesity and insulin resistance in mice fed a high-fat diet and in non-human primates with spontaneous obesity (Macia *et al.* 2012, Tsai *et al.* 2013, Chrysovergis *et al.* 2014, Mullican *et al.* 2017), while GDF15 knockout mice were more susceptible to obesity induced by a high-fat diet, exhibited worse glucose tolerance, and had a lower metabolic rate compared to wildtype mice (Tran *et al.* 2018). When this protein is administered to rodents and non-human primates, it significantly reduced their body weights; however, this effect was mainly attributed to a decrease in food intake (Emmerson *et al.* 2017, Xiong *et al.* 2017, Tsai *et al.* 2018), although an increase in energy expenditure may also contribute (Tsai *et al.* 2018, Wang *et al.* 2023). These effects are mediated by the glial cell line-derived neurotrophic factor (GDNF) family receptor α-like (GFRAL), the GDF15 receptor, which is expressed in the hindbrain, specifically in the area postrema (AP) and the nucleus of the solitary tract (NTS) (Emmerson *et al.* 2017, Hsu *et al.* 2017, Mullican *et al.* 2017, Yang *et al.* 2017, Suriben *et al.* 2020, Sjoberg *et al.* 2023). It was suggested that glucagon-like peptide-1 (GLP-1) and GDF15 could act through the same mechanisms, but combined treatments in rodents showed synergistic effects, indicating distinct pathways (Emmerson *et al.* 2017, Frikke-Schmidt *et al.* 2019, Ghidewon *et al.* 2022). A dual GLP-1/GDF15 agonist led to greater reductions in body weight, food intake, insulin, fasting glucose, and triglycerides compared to individual treatments (Zhang *et al.* 2023*b*), highlighting GDF15’s potential in obesity management.

Bariatric surgery (BS) has been widely recognized in recent years as an effective treatment for obesity and related complications, such as type T2D (Yang *et al.* 2024). Several studies have reported an increase in circulating levels of GDF15 after BS, with some of them suggesting that surgery-induced weight loss is related to increased circulating GDF15 values (Vila *et al.* 2011, Kleinert *et al.* 2019, Dolo *et al.* 2020, Salman *et al.* 2021). Di Vincenzo *et al.* observed that sleeve gastrectomy (SG) preferentially increased GDF15 plasma levels in patients with obesity who did not present metabolic syndrome, highlighting a differential response based on metabolic phenotype (Di Vincenzo *et al.* 2025). In contrast, Chaiyasoot *et al.* observed that, after 1 year of pharmacological (topiramate) or BS treatments for weight loss, a decrease in circulating GDF15 levels was associated with greater weight loss (Chaiyasoot *et al.* 2023). Other studies have not found differences in circulating GDF15 values between patients with or without BS (Martinussen *et al.* 2021). These discrepancies highlight the complexity of GDF15 regulation and its relationship to metabolic status.

Given that circulating levels of GDF15 can be influenced by various factors including surgery type, sex, age, presence of metabolic syndrome, and medication (Asrih *et al.* 2023, Li *et al.* 2024*a*, Dong & Xu 2024), accurately characterizing the study population is essential to fully understand the role of circulating GDF15 levels in BS outcomes. Therefore, the objective of this study was to assess circulating GDF15 levels in a cohort of patients with obesity and a cohort of healthy, normal-weight individuals. Patients with obesity were evaluated before BS and at 3, 6, and 12 months post-surgery. Patients with obesity were evaluated before and 3, 6, and 12 months after BS, and circulating GDF15 levels were analysed in relation to clinical, biochemical, and anthropometric parameters.

## Materials and methods

### Patients and sample collection

This study was approved by the Research Ethics Committee of Galicia, Spain (references 2014/135 and 2022/213), in accordance with the Declaration of Helsinki-II. Written informed consent was obtained from all participants.

The inclusion criteria for patients who underwent BS were: 18–65 years of age at the time of surgery, a BMI >40 kg/m^2^ or a BMI >35 kg/m^2^ (if they have high-risk comorbidities, such as HBP, diabetes, OSA or cardiovascular risk factors), a history of failures in previous nonsurgical attempts to lose weight, and expectation that the patient will comply with postoperative care and follow-up visits. The exclusion criteria were: serious psychiatric illnesses, drug or alcohol abuse, and lack of understanding of the benefits, risks, alternatives, expected results and lifestyle changes necessary after BS. To ensure that all candidates for BS met the established criteria, the candidates were evaluated by a team made up of a digestive surgeon, an endocrinologist and a psychiatrist. Based on the clinical characteristics of each patient (BMI, age and health problems), they were assigned either SG or Roux-en-Y gastric bypass (RYGB).

Fasting blood samples were collected from normal-weight, healthy individuals (*n* = 72) and patients with obesity before BS (*n* = 144) and 3 (*T* = 3, *n* = 64), 6 (*T* = 6, *n* = 71) and 12 (*T* = 12, *n* = 106) months after BS, either RYGB or SG. The blood was collected in serum gel separator tubes. Within 1 h of the blood draw, tubes were centrifuged at 4,000 *g* for 15 min. The serum was transferred to polypropylene tubes and stored at −80°C until analysis.

### Blood parameter analysis

Circulating GDF15 levels were analysed in serum samples using an ELISA kit, according to the manufacturer's instructions (Invitrogen, Spain; reference EHGDF15). The analytical sensitivity of the assay was 2 pg/mL. The intra-assay coefficient of variation was <10%, and the inter-assay coefficient of variation was <12%.

The remaining blood data shown in this study were taken from the volunteers’ medical records.

### Body composition analysis

Body composition was analysed using bioelectrical impedance analysis (BIA) with a tetrapolar bioimpedantiometer BC-418 segmental body composition analyser (TANITA).

### Calculations

BMI was calculated using the following formula: weight (kg)/height (m)^2^.

% Excess body mass index lost (EBMIL) was calculated using the formula: ((preoperative BMI − current BMI)/(preoperative BMI − 25)) × 100.

The homoeostasis model assessment as an index of insulin resistance (HOMA-IR) was calculated using the formula: [(((fasting glucose (mg/dL) × fasting insulin (μU/mL))/405].

% Excess weight lost (EWL) was calculated using the formulae: for women = [((preoperative weight (kg) − current weight (kg))/(preoperative weight (kg) − (54.09 + (((height (cm) − 152)/2.54) × 1.36)))) × 100] and for men = [((preoperative weight (kg) − current weight (kg))/(preoperative weight (kg) − (61.36 + (((height (cm) − 159.6)/2.54) × 1.36)))) × 100].

### Data analysis

Continuous variables are expressed as mean ± standard error of the mean (SEM). Categorical data are expressed as frequency and percentages. For comparisons between control subjects and patients with obesity, unpaired Student’s *T*-test was used for normally distributed data and the Mann–Whitney U test was used for non-normally distributed variables for comparison of medians. For categorical comparisons, the χ2 test was used. For the contrast of repeated measures (before BS and 12 months after BS), Wilcoxon signed-rank test was used. For comparisons between patients with obesity before and after BS, two-way ANOVA with Tukey’s post hoc test was used. Spearman correlations were computed to assess the relationship between variables. Linear mixed models were used to investigate the variation of circulating GDF15 levels in patients with obesity undergoing BS. Assuming that circulating GDF15 levels decreased exponentially, the natural log-transformed titres were modelled over time. Time was expressed in months since surgery. Correlated random intercepts and slopes were allowed. The univariate effect of covariates, including age, sex, gender, type of surgery, presence of T2D and HBP and metformin treatment, on the intercept and slope of the fixed effect was analysed. A multivariable model including all analysed covariates was conducted to study the independent effects on the reduction of circulating GDF15 levels.

Statistics were performed with GraphPad Prism, version 9.0, and SPSS, version 24.0. *P*-values <0.05 were considered as statistically significant.

## Results

Fasting circulating levels of GDF15 were measured in patients with obesity (obese group) both before and at 3, 6, and 12 months after BS. Samples from healthy, normal-weight individuals (control group) were also analysed. The anthropometric, clinical and biochemical parameters of control group and patients with obesity before BS are presented in Table 1. In patients who underwent BS, the progression of these parameters at 3, 6, and 12 months post-surgery is presented in Table 2.

**Table 1:** Clinical, biochemical and metabolic characteristics of healthy, normal-weight individuals (control group) and patients with obesity (obese group) included in this study.

		Control	Obese	*P* value (control vs obese)
Sex (*n*)	Men	32	60	
	Women	40	84	
Age (years)	Men	42.98 ± 1.89	48.49 ± 1.26	0.01
	Women	41.93 ± 1.29	47.08 ± 1.10	0.006
T2D (%)	Men	0	55.00	<0.001
	Women	0	22.62***	0.003
Steatosis (%)	Men	-	85.00	
	Women	-	59.52**	
HBP (%)	Men	0	76.67	<0.001
	Women	0	44.05***	<0.001
OSA (%)	Men	0	30.00	0.016
	Women	0	19.05	0.008
ADD (%)	Men	6.25	35.00	0.201
	Women	2.50	50.00	<0.001
DL (%)	Men	0	50.00	0.002
	Women	0	26.19**	<0.001
BMI (kg/m^2^)	Men	24.24 ± 0.43	48.20 ± 1.06	<0.001
	Women	22.98 ± 0.57	48.22 ± 0.57	<0.001
Fat mass (%)	Men	19.08 ± 0.84	41.51 ± 0.79	<0.001
	Women	28.33 ± 1.48***	51.90 ± 0.55***	<0.001
Glucose (mg/dL)	Men	92.65 ± 1.81	99.35 ± 5.04	0.519
	Women	85.07 ± 1.01	92.71 ± 1.81	0.208
Insulin (μUI/mL)	Men	4.08 ± 0.61	8.42 ± 1.17	0.046
	Women	5.23 ± 0.88	6.32 ± 0.54*	0.214
C-peptide (ng/mL)	Men	1.74 ± 0.36	2.89 ± 0.23	<0.001
	Women	1.55 ± 0.14	2.29 ± 0.14*	<0.001
HbA1c (%)	Men	5.18 ± 0.07	5.98 ± 0.15	<0.001
	Women	5.04 ± 0.07	5.65 ± 0.13	0.001
HOMA-IR	Men	0.97 ± 0.17	2.44 ± 0.51	0.085
	Women	1.14 ± 0.22	1.51 ± 0.15	0.176
Urea (mg/dL)	Men	37.67 ± 2.09	36.46 ± 2.87	0.130
	Women	31.14 ± 2.09*	30.55 ± 1.66**	0.328
Creatinine (mg/dL)	Men	0.96 ± 0.03	0.93 ± 0.07	0.653
	Women	0.77 ± 0.03***	0.68 ± 0.03***	0.001
Decreased GFR (%)	Men	9.38	15.00	0.674
	Women	5.00	14.29	0.345
Uric acid (mg/dL)	Men	5.76 ± 0.28	7.08 ± 0.32	0.023
	Women	3.93 ± 0.13***	6.30 ± 0.26*	<0.001
Albumin (g/dL)	Men	4.57 ± 0.10	3.94 ± 0.08	<0.001
	Women	4.57 ± 0.06	3.92 ± 0.08	<0.001
Total bilirubin (mg/dL)	Men	0.72 ± 0.09	0.71 ± 0.06	0.644
	Women	0.67 ± 0.10	0.52 ± 0.03**	0.164
Total cholesterol (mg/dL)	Men	199.45 ± 6.40	131.64 ± 6.59	<0.001
	Women	194.13 ± 6.48	143.88 ± 4.22	<0.001
HDL cholesterol (mg/dL)	Men	56.00 ± 2.21	26.71 ± 0.79	<0.001
	Women	64.54 ± 2.34***	32.64 ± 0.95***	<0.001
LDL cholesterol (mg/dL)	Men	124.19 ± 5.47	79.31 ± 6.18	<0.001
	Women	116.74 ± 5.80	89.60 ± 3.16	<0.001
Triglycerides (mg/dL)	Men	96.14 ± 8.14	149.40 ± 16.65	<0.001
	Women	72.07 ± 4.52**	114.79 ± 5.97**	<0.001
ApoA (mg/dL)	Men	170.43 ± 6.69	101.45 ± 3.60	<0.001
	Women	173.34 ± 5.83	110.13 ± 2.86	<0.001
ApoB (mg/dL)	Men	109.13 ± 5.44	88.05 ± 5.78	<0.001
	Women	98.23 ± 5.54	84.91 ± 2.86	0.026
ALP (UI/L)	Men	103.75 ± 22.47	117.40 ± 7.69	0.716
	Women	102.33 ± 12.91	83.86 ± 5.09	0.184
AST (UI/L)	Men	23.63 ± 1.27	38.70 ± 3.17	<0.001
	Women	18.97 ± 0.95***	34.94 ± 2.14	<0.001
ALT (UI/L)	Men	25.68 ± 2.26	47.94 ± 4.39	<0.001
	Women	17.59 ± 1.57***	43.06 ± 3.06	<0.001
GGT (UI/L)	Men	28.83 ± 6.12	28.18 ± 2.27	0.728
	Women	12.80 ± 1.03**	29.72 ± 3.94	<0.001
CRP (mg/dL)	Men	0.14 ± 0.06	0.97 ± 0.17	<0.001
	Women	0.13 ± 0.04	1.04 ± 0.11	<0.001

Data are expressed as mean ± standard error of the mean (SEM) or percentage (%). Abbreviations: T2D, type 2 diabetes; HBP, high blood pressure; OSA, obstructive sleep apnoea; ADD, anxiety-depressive disorder; DL, dyslipidaemia; GFR, glomerular filtration rate; ALP, alkaline phosphatase; AST, aspartate aminotransferase; ALT, alanine aminotransferase; GGT, gamma-glutamyl transferase; ApoA, apolipoprotein A; ApoB, apolipoprotein B; HbA1c, glycated haemoglobin; CRP, C-reactive protein; HOMA-IR, homoeostatic model assessment for insulin resistance; -, no data available. *T*-test comparing groups, reflecting the significance of an independent sample *T*-test for normally distributed variables or a Mann–Whitney U test for non-normally distributed variables (quantitative variables) or Chi-square test (qualitative variables). *, **, ***: *P* < 0.05, *P* < 0.01, *P* < 0.001 men vs women within the control/obese group.

**Table 2:** Clinical, biochemical and metabolic characteristics of patients with obesity before BS (obese T0) and 3 (obese T3), 6 (obese T6) and 12 (obese T12) months after BS.

		Obese T0	Obese T3	Obese T6	Obese T12
Sex (*n*)	Men	60	20	18	43
	Women	84	44	53	63
Age (years)	Men	48.49 ± 1.26	48.47 ± 2.11	49.35 ± 2.79	48.57 ± 1.47
	Women	47.08 ± 1.10	46.31 ± 1.43	47.74 ± 1.31	48.35 ± 1.21
T2D (%)	Men	55.00^a^	5.00^b^	11.11^b^	9.30^b^
	Women	22.62^a,^***	2.27^b^	3.77^b^	3.17^b^
Steatosis (%)	Men	85.00	-	-	-
	Women	59.52**	-	-	-
HBP (%)	Men	76.67^a^	20.00^b^	38.89^b^	34.88^b^
	Women	44.05^a,^***	15.91^b^	22.64^b^	19.05^b^
OSA (%)	Men	30.00	-	-	-
	Women	19.05	-	-	-
ADD (%)	Men	35.00	25.00	27.78	20.93
	Women	50.00^a^	36.36^a,b^	41.51^a^	22.22^b^
DL (%)	Men	50.00^a^	35.00^a^	22.22^a,b^	16.28^b^
	Women	26.19^a,^**	4.55^b,^**	11.32^b^	7.94^b^
BMI (kg/m^2^)	Men	48.20 ± 1.06^a^	38.95 ± 1.88^b^	33.69 ± 1.04^b,c^	32.69 ± 0.83^c^
	Women	48.22 ± 0.57^a^	39.26 ± 1.05^b^	35.84 ± 1.06^b^	31.32 ± 0.74^c^
Fat mass (%)	Men	41.51 ± 0.79^a^	32.44 ± 1.58^b^	28.52 ± 2.09^b^	27.71 ± 1.43^b^
	Women	51.90 ± 0.55^a,^***	46.70 ± 0.79^b,^***	40.70 ± 1.02^c,^***	34.70 ± 1.17^d,^***
EWL (%)	Men		41.24 ± 2.55^a^	54.36 ± 2.51^b^	60.35 ± 2.43^b^
	Women		36.80 ± 1.56^a^	52.39 ± 1.96^b^	66.90 ± 2.36^c,^*
EBMIL (%)	Men		46.41 ± 3.20^a^	61.03 ± 3.06^b^	67.72 ± 2.88^b^
	Women		40.66 ± 1.77^a^	58.49 ± 2.54^b^	74.46 ± 2.69^c^
Glucose (mg/dL)	Men	99.35 ± 5.04^a^	93.25 ± 4.45^a,b^	96.65 ± 5.22^a,b^	86.93 ± 1.74^b^
	Women	92.71 ± 1.81^a^	84.95 ± 1.75^a,b^	83.88 ± 1.88^a,b,^*	83.40 ± 1.41^b^
Insulin (μUI/mL)	Men	8.42 ± 1.17	9.51 ± 1.55	9.63 ± 1.43	7.40 ± 0.79
	Women	6.32 ± 0.54*	8.10 ± 0.70	6.03 ± 0.52*	5.46 ± 0.51
C-peptide (ng/mL)	Men	2.89 ± 0.23	2.64 ± 0.21	2.57 ± 0.23	2.47 ± 0.15
	Women	2.29 ± 0.14^a,b,^**	2.52 ± 0.13^a^	1.95 ± 0.08^b,^*	1.77 ± 0.08^c,^***
HbA1c (%)	Men	5.98 ± 0.15	5.39 ± 0.17	5.66 ± 0.35	5.26 ± 0.11
	Women	5.65 ± 0.13	5.77 ± 0.55	5.21 ± 0.07	5.21 ± 0.07
HOMA-IR	Men	2.39 ± 0.50	2.14 ± 0.34	2.34 ± 0.40	1.62 ± 0.19
	Women	1.51 ± 0.15**	1.67 ± 0.16	1.27 ± 0.11*	1.14 ± 0.11
Urea (mg/dL)	Men	36.46 ± 2.87	31.35 ± 3.19	31.65 ± 2.28	40.93 ± 3.65
	Women	30.55 ± 1.66*	27.55 ± 1.16	30.92 ± 1.19	34.72 ± 1.23*
Creatinine (mg/dL)	Men	0.93 ± 0.07	0.84 ± 0.06	0.82 ± 0.04	0.93 ± 0.11
	Women	0.68 ± 0.03***	0.65 ± 0.02*	0.64 ± 0.02	0.63 ± 0.01***
Decreased GFR (%)	Men	15.00	5.00	5.56	9.30
	Women	14.29	18.80	13.21	9.52
Uric acid (mg/dL)	Men	7.08 ± 0.32^a^	6.31 ± 0.24^a,b^	5.67 ± 0.32^b^	5.96 ± 0.22^b^
	Women	6.30 ± 0.26^a,^*	5.43 ± 0.20^a,b^	5.04 ± 0.18^b^	4.57 ± 0.31^b,^***
Albumin (g/dL)	Men	3.94 ± 0.08^a^	4.45 ± 0.08^b^	4.45 ± 0.06^b^	4.39 ± 0.07^b^
	Women	3.92 ± 0.08^a^	4.34 ± 0.04^b^	4.28 ± 0.03^b^	4.26 ± 0.03^b^
Total bilirubin (mg/dL)	Men	0.71 ± 0.06	0.87 ± 0.07	0.97 ± 0.15	0.91 ± 0.10
	Women	0.52 ± 0.03**	0.68 ± 0.03	0.65 ± 0.03**	0.62 ± 0.03***
Total cholesterol (mg/dL)	Men	131.64 ± 6.59^a^	157.00 ± 6.67^a,b^	155.29 ± 10.67^a,b^	174.60 ± 6.63^b^
	Women	143.88 ± 4.22^a^	184.82 ± 5.76^b,^**	180.17 ± 4.72^b,^*	181.87 ± 4.67^b^
HDL cholesterol (mg/dL)	Men	26.71 ± 0.79^a^	38.32 ± 2.23^b^	41.94 ± 2.87^b,c^	47.69 ± 1.50^c^
	Women	32.64 ± 0.95^a,^***	42.86 ± 1.29^b^	49.37 ± 1.17^c,^**	58.65 ± 1.30^d,^***
LDL cholesterol (mg/dL)	Men	79.31 ± 6.18^a^	98.04 ± 6.42^a,b^	98.23 ± 8.84^a,b^	106.52 ± 5.58^b^
	Women	89.60 ± 3.16^a^	120.49 ± 4.78^b,^*	111.50 ± 3.83^b^	106.37 ± 3.89^b^
Triglycerides (mg/dL)	Men	149.40 ± 16.65^a^	108.5 ± 4.75^b^	98.06 ± 6.47^b^	95.43 ± 5.91^b^
	Women	114.79 ± 5.97^a,^***	114.34 ± 4.99^a^	98.02 ± 4.11^a,b^	84.45 ± 4.39^b^
ApoA (mg/dL)	Men	101.45 ± 3.60^a^	128.23 ± 5.88^b^	136.15 ± 6.65^b^	147.19 ± 4.33^b^
	Women	110.13 ± 2.86^a,^*	127.63 ± 2.89^b^	144.53 ± 3.06^c^	161.49 ± 2.82^d,^**
ApoB (mg/dL)	Men	88.05 ± 5.78	83.33 ± 5.97	80.85 ± 7.00	96.74 ± 4.95
	Women	84.91 ± 2.86^a^	106.66 ± 3.73^b,^*	100.16 ± 3.55^b,^*	95.26 ± 3.60^a,b^
ALP (UI/L)	Men	117.40 ± 7.69	135.8 ± 14.26	129.18 ± 13.52	123.40 ± 11.36
	Women	83.86 ± 5.09***	82.50 ± 5.12***	84.02 ± 4.86***	87.24 ± 6.30***
AST (UI/L)	Men	38.70 ± 3.17^a^	23.32 ± 2.11^b^	20.71 ± 1.94^b^	21.61 ± 0.78^b^
	Women	34.94 ± 2.14^a^	20.72 ± 0.96^b^	22.82 ± 1.58^b^	23.34 ± 1.31^b^
ALT (UI/L)	Men	47.94 ± 4.39^a^	24.55 ± 2.78^b^	21.29 ± 2.87^b^	23.20 ± 1.44^b^
	Women	43.06 ± 3.06^a^	22.75 ± 1.83^b^	23.90 ± 2.81^b^	28.93 ± 3.96^b^
GGT (UI/L)	Men	28.18 ± 2.27	23.65 ± 2.75	25.41 ± 6.41	34.48 ± 9.84
	Women	29.72 ± 3.94	20.02 ± 3.78	19.88 ± 3.56	18.25 ± 2.20
CRP (mg/dL)	Men	0.97 ± 0.17^a^	0.50 ± 0.19^a,b^	0.35 ± 0.18^b^	0.15 ± 0.04^b^
	Women	1.04 ± 0.11^a^	0.61 ± 0.10^b^	0.47 ± 0.10^b,c^	0.17 ± 0.04^c^

Data are expressed as mean ± standard error of the mean (SEM) or percentage (%). Abbreviations: T2D, type 2 diabetes; HBP, high blood pressure; OSA, obstructive sleep apnoea; ADD, anxiety-depressive disorder; DL, dyslipidaemia; GFR, glomerular filtration rate; EWL, excess weight lost; EBMIL, excess body mass index lost; ALP, alkaline phosphatase; AST, aspartate aminotransferase; ALT, alanine aminotransferase; GGT, gamma-glutamyl transferase; ApoA, apolipoprotein A; ApoB, apolipoprotein B; HbA1c, glycated haemoglobin; CRP, C-reactive protein; HOMA-IR, homoeostatic model assessment for insulin resistance; -, no data available; BS, bariatric surgery.

Two-way ANOVA with Tukey’s post hoc (quantitative variables) or Chi-square test (qualitative variables). Within each sex, different letters indicate statistically significant differences over time. *, **, ***: *P* < 0.05, *P* < 0.01, *P* < 0.001 men vs women within the same time point.

**Figure 1:**
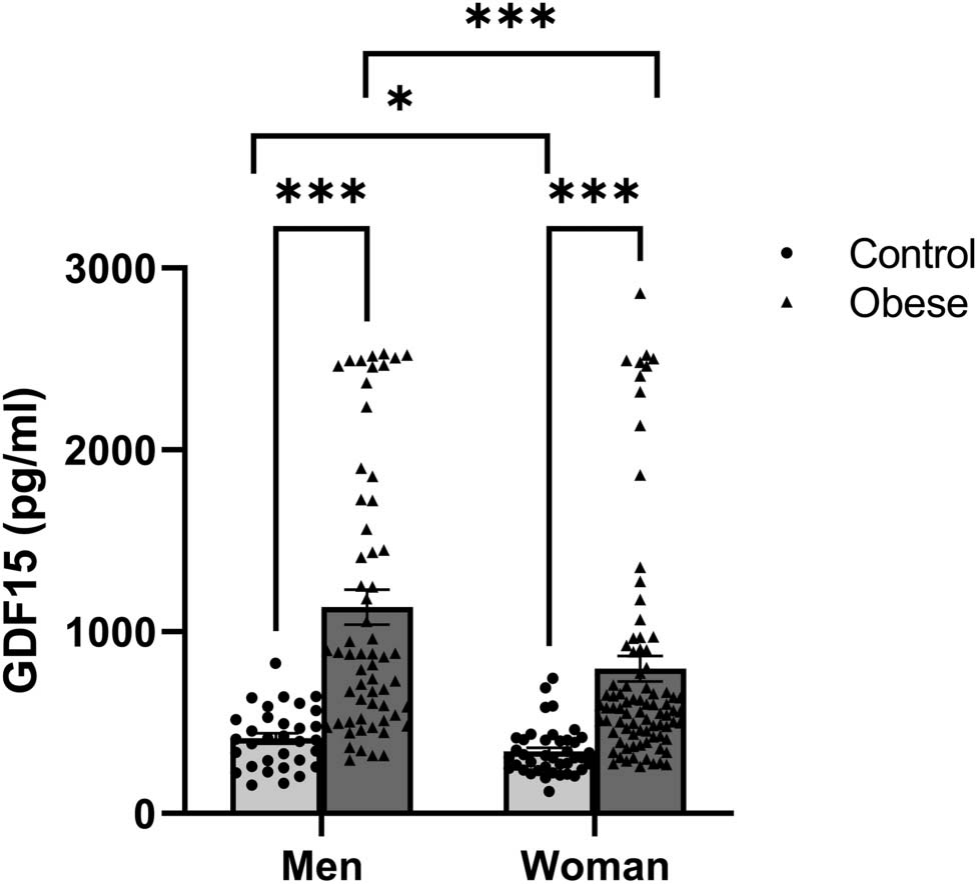
Serum GDF15 levels in normal-weight individuals (control group) and patients with obesity (obese group). Data are expressed as mean ± standard error of mean (SEM). *, ***P < 0.05, *P* < 0.001 by Mann–Whitney U test. Control group: men *n* = 32 and women *n* = 40; obese group: men *n* = 60 and women *n* = 84.

**Figure 2:**
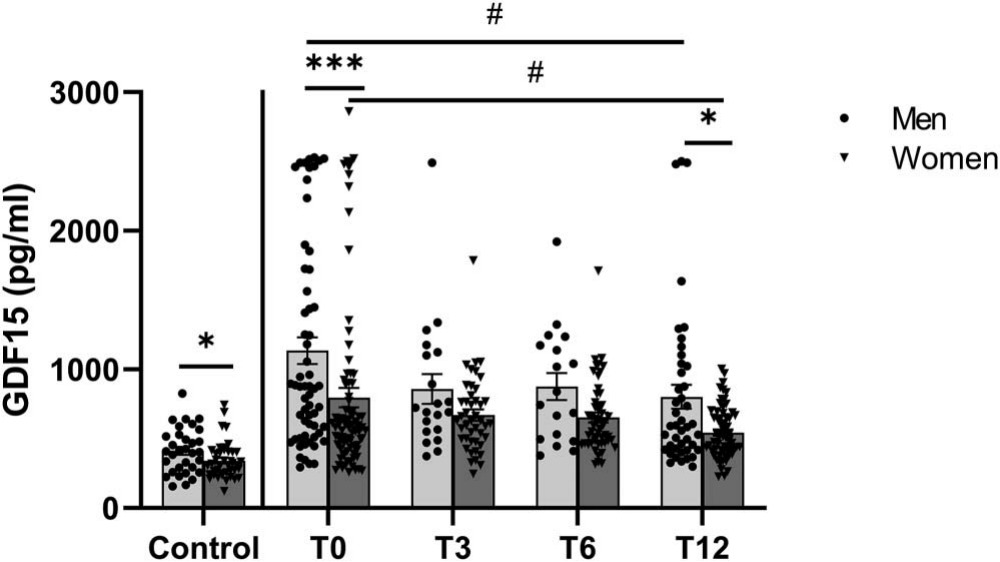
Serum GDF15 levels in normal-weight individuals (control group) and patients with obesity before bariatric surgery (T0) and at 3 (T3), 6 (T6) and 12 (T12) months. Data are expressed as mean ± standard error of mean (SEM). Comparisons within the control group were carried out with Mann–Whitney U test, *P < 0.05 male vs female. Two-way ANOVA with Tukey’s post hoc was used to study the evolution of obese patients after bariatric surgery. ^#^*P* < 0.05 T0 vs T12 within the same sex. *, ***P < 0.05, *P* < 0.001 male vs female within the same time point. T0: obese patients before bariatric surgery, T3: obese patients 3 months after bariatric surgery, T6: obese patients 6 months after bariatric surgery, T12: obese patients 12 months after bariatric surgery. Control group: men *n* = 32 and women *n* = 40; T0 group: men *n* = 60 and women *n* = 84; T3 group: men *n* = 20 and women *n* = 44; T6 group: men *n* = 18 and women *n* = 53; T12 group: men *n* = 43 and women *n* = 63.

Figure 1 shows that circulating GDF15 levels increased with obesity in both men and women, with men consistently exhibiting the highest values. Figure 2 illustrates a progressive decrease in circulating GDF15 concentrations after BS, reaching statistically significant differences 1 year after surgery. Despite this significant decrease, circulating GDF15 values remained higher than that in the control group for both sexes (*P* < 0.001, statistic not shown). Given that women exhibited lower circulating levels of GDF15 compared to men, we subsequently examined whether the reduction in circulating GDF15 levels 1 year after surgery differed by sex. The results, both overall and stratified by sex, are presented in Table 3. As shown, both men and women experienced similar reductions in circulating GDF15 levels 1 year post-surgery.

**Table 3:** Serum GDF15 levels and ΔGDF15 before (T0) and 12 months after (T12) bariatric surgery.

		Mean ± SEM (pg/mL)	*n*	*P* value
GDF15 T0	Global (men + women)	904.09 ± 65.23	106	0.001*
GDF15 T12	Global (men + women)	653.71 ± 39.57	106	
ΔGDF15 (T12–T0)	Global (men + women)	−250.37 ± 57.76	106	
GDF15 T0	Men	1,135.17 ± 95.75	60	<0.001^†^
	Women	797.25 ± 69.85	84	
GDF15 T12	Men	801,63 ± 86.30	43	0.059^†^
	Women	552.75 ± 24.76	63	
ΔGDF15 (T12–T0)	Men	−300.84 ± 101.27	43	0.107^†^
	Women	−215.93 ± 68.68	63	

* *P*-value for the contrast of repeated measures using the Wilcoxon test.

^†^ *P*-value for the contrast of independent measures, according to sex, using the Mann–Whitney U test. SEM, standard error of the mean.

To assess whether circulating GDF15 levels are related to anthropometric parameters, we analysed the Spearman correlation coefficients of circulating GDF15 levels with weight, BMI, and % fat mass. This analysis was performed both for the combined groups of patients with obesity and normal-weight and exclusively for the cohort of patients with obesity. The results are shown in Table 4, where it can be observed that circulating GDF15 levels exhibit positive correlations with weight, BMI, and body fat percentage when both cohorts are analysed together. However, when analysing only the cohort of patients with obesity, circulating GDF15 levels maintain positive correlations with weight and BMI, but showed a negative correlation with body fat percentage, indicating that GDF15 levels are inversely associated with adiposity within the obese population, suggesting that elevated circulating GDF15 levels may confer some protection against fat accumulation in individuals with obesity. We further examined the evolution of these correlation coefficients in the cohort of patients with obesity after undergoing BS. In addition, we also studied whether the changes in circulating GDF15 levels (ΔGDF15) after surgery correlate with any of the anthropometric parameters analysed in this work. At 3 months post-BS, ΔGDF15 showed a statistically significant positive correlation with weight loss (with BMI *P* = 0.05). One year post-surgery, a negative correlation with BMI and positive correlations with % EWL and % EBMIL were observed (Table 4).

**Table 4:** Spearman correlations of different anthropometric parameters with circulating GDF15 values and changes in circulating GDF15 levels (ΔGDF15).

	Spearman rho correlation coefficient	*P* value	*n*
**Cohorts of patients with obesity before BS + control patients**			
GDF15 – weight	**0.519**	**<0.001**	**216**
GDF15 – BMI	**0.532**	**<0.001**	**216**
GDF15 – % fat mass	**0.335**	**<0.001**	**148**
***T* = 0 (cohort of patients with obesity before BS)**			
GDF15 – weight	**0.169**	**0.043**	144
GDF15 – BMI	**0.184**	**0.028**	144
GDF15 – % fat mass	**−0.256**	**0.011**	97
***T* = 3 (cohort of patients with obesity 3 months after BS)**			
GDF15 – weight	0.148	0.246	63
GDF15 – weight loss	−0.053	0.681	63
GDF15 – BMI	0.095	0.458	63
GDF15 – BMI loss	−0.001	0.992	63
GDF15 – % fat mass	−0.047	0.725	59
GDF15 – % fat mass loss	−0.023	0.893	38
GDF15 – % EWL	−0.070	0.583	63
GDF15 – % EBMIL	−0.068	0.599	63
ΔGDF15 – weight	−0.163	0.203	63
ΔGDF15 – weight loss	**0.271**	**0.032**	63
ΔGDF15 – BMI	−0.071	0.581	63
ΔGDF15 – BMI loss	0.248	0.050	63
ΔGDF15 – % fat mass	0.165	0.213	59
ΔGDF15 – % fat mass loss	0.183	0.271	38
ΔGDF15 – % EWL	−0.071	0.580	63
ΔGDF15 – % EBMIL	−0.041	0.749	63
***T* = 6 (cohort of patients with obesity 6 months after BS)**			
GDF15 – weight	−0.031	0.800	70
GDF15 – weight loss	−0.010	0.933	70
GDF15 – BMI	−0.055	0.654	70
GDF15 – BMI loss	−0.034	0.777	70
GDF15 – % fat mass	−0.169	0.181	64
GDF15 – % fat mass loss	0.000	0.999	40
GDF15 – % EWL	0.044	0.718	70
GDF15 – % EBMIL	0.036	0.770	70
ΔGDF15 – weight	−0.102	0.432	62
ΔGDF15 – weight loss	−0.222	0.083	62
ΔGDF15 – BMI	−0.201	0.117	62
ΔGDF15 – BMI loss	−0.229	0.074	62
ΔGDF15 – % fat mass	−0.223	0.089	59
ΔGDF15 – % fat mass loss	−0.324	0.058	35
ΔGDF15 – % EWL	0.204	0.111	62
ΔGDF15 – % EBMIL	0.0227	0.076	62
***T* = 12 (cohort of patients with obesity 12 months after BS)**			
GDF15 – weight	0.032	0.750	104
GDF15 – weight loss	−0.037	0.712	104
GDF15 – BMI	0.006	0.952	104
GDF15 – BMI loss	−0.013	0.893	104
GDF15 – % fat mass	−0.014	0.894	99
GDF15 – % fat mass loss	−0.014	0.913	65
GDF15 – % EWL	0.029	0.772	104
GDF15 – % EBMIL	0.028	0.774	104
ΔGDF15 – weight	−0.173	0.079	104
ΔGDF15 – weight loss	−0.044	0.658	104
ΔGDF15 – BMI	**−0.235**	**0.016**	104
ΔGDF15 – BMI loss	−0.042	0.674	104
ΔGDF15 – % fat mass	−0.153	0.130	99
ΔGDF15 – % fat mass loss	0.082	0.518	65
ΔGDF15 – % EWL	**0.212**	**0.031**	104
ΔGDF15 – % EBMIL	**0.238**	**0.015**	104

Bold indicates statistical significance. BS, bariatric surgery; BMI, body mass index; EBMIL, excess body mass index lost; EWL, excess weight lost.

When data from controls and individuals with obesity were taken together, circulating GDF15 levels were found to positively correlate with several factors related to glucose metabolism (glucose, insulin, C-peptide, HbA1c and HOMA-IR), liver health (AST, ALT and GGT), kidney health (urea, creatinine and uric acid) and inflammatory markers (CRP). In contrast, they were negatively correlated with parameters associated with lipid metabolism (total cholesterol, HDL-cholesterol, LDL-cholesterol and apolipoprotein A), except for total triglyceride levels, which showed a positive correlation (see Supplementary Table 1 (see section on Supplementary materials given at the end of the article)). In the cohort of individuals with obesity, most of the above correlations remained significant before BS. After BS, especially after 1 year, some of the previously mentioned parameters continued to correlate with circulating GDF15 levels, although in most cases, the correlations were weaker. Among the factors that maintained a correlation, the following were particularly notable: glucose, HbA1c, triglycerides, urea, creatinine, and uric acid (see Supplementary Table 1).

Next, we aimed to investigate whether the absence or presence of T2D and HBP, and the type of BS, influenced ΔGDF15 1 year after BS. The results are presented in Table 5. Patients with HBP or T2D at the time of surgery exhibited statistically significant changes in circulating GDF15 levels 1 year post-surgery, regardless of whether the data were analysed collectively for men and women, or disaggregated by sex. However, patients without T2D or HBP did not exhibit this difference. Overall, both SG and RYGB showed significant decreases after 1 year. However, when analysed by sex, only the SG group maintained statistical significance. In addition, preoperative circulating GDF15 levels were lower in women undergoing SG compared to those undergoing RYGB (see Supplementary Table 2), likely reflecting the distinct clinical characteristics of women referred for each type of surgery.

**Table 5:** Changes in serum GDF15 levels (ΔGDF15) from preoperative to 1 year after BS based on type of surgery, absence or presence of T2D and presence or absence of HBP.

	Mean ± SEM	*n*	*P*	Mean ± SEM	*n*	*P*
	**SG**			**RYGB**		
Global (men + women)	−220.6 ± 75.1	51	<0.001	−278.0 ± 87.0	55	0.012
Men	−324.4 ± 139.7	24	0.006	−271.1 ± 150.1	19	0.084
Women	−128.3 ± 68.4	27	0.037	−281.6 ± 108.3	36	0.093
	**No T2D**			**Yes T2D**		
Global (men + women)	−76.4 ± 4.6	71	0.054	−603.3 ± 124.4	35	<0.001
Men	−120.8 ± 143.1	21	0.122	−472.7 ± 137.4	22	0.002
Women	−57.7 ± 38.1	50	0.211	−824.5 ± 237.6	13	0.011
	**No HBP**			**Yes HPB**		
Global (men + women)	−105.5 ± 55.9	46	0.114	−361.5 ± 90.4	60	<0.001
Men	−20.2 ± 67.8	10	0.386	−385.9 ± 127.2	33	0.001
Women	−129.1 ± 68.8	36	0.220	−331.6 ± 129.8	27	0.021

BS, bariatric surgery; HPB, high blood pressure; RYGB, Roux-en-Y gastric bypass; SG, sleeve gastrectomy; T2D, type 2 diabetes. *P*-values are shown for the contrast of repeated measures using the Wilcoxon test, globally and by sex, according to the type of surgery, absence or presence of type 2 diabetes and presence or absence of arterial hypertension.

**Figure 3:**
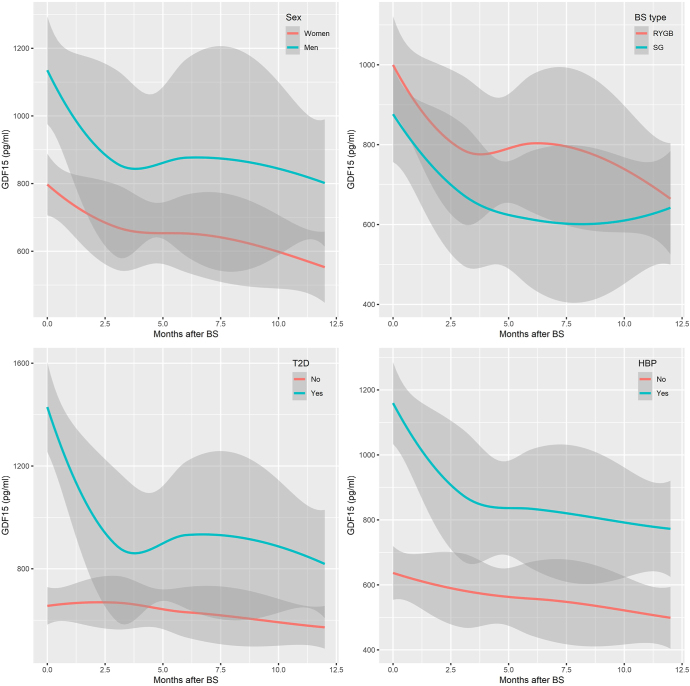
Graphical representation of linear mixed models. The mean trajectories and 95% confidence intervals are shown as solid lines and shaded areas, respectively. BS, bariatric surgery; HBP, high blood pressure; RYGB, Roux-en-Y gastric bypass; SG, sleeve gastrectomy; T2D, type 2 diabetes.

Following this line of investigation and using linear mixed models, we analysed the evolution of circulating GDF15 levels over 1 year after BS, considering the presence or absence of T2D and HBP, and sex and type of surgery (Fig. 3). Men consistently displayed higher circulating GDF15 levels than women, with both sexes showing a significant post-surgical decrease, and men experiencing more pronounced reductions and variability. Patients undergoing SG initially exhibited a greater reduction in circulating GDF15 levels than those undergoing RYGB, although levels stabilised after 6 months, with SG patients maintaining slightly lower levels. Diabetic and hypertensive patients had higher baseline circulating GDF15 levels and experienced a marked reduction in the early months post-surgery. In contrast, non-diabetic and non-hypertensive patients had lower initial circulating GDF15 levels, undergoing a more gradual decrease over time. By the end of the follow-up period, non-hypertensive patients maintained lower circulating GDF15 levels, while hypertensive patients exhibited greater variability in their levels.

Table 6 presents the results of both univariable and multivariable linear mixed models used to estimate circulating GDF15 values. In the univariable model, age, sex, diabetes, hypertension, and metformin treatment were found to significantly affect circulating GDF15 levels. However, in the multivariable model, after adjusting for all other variables, only hypertension remained a statistically significant predictor of circulating GDF15 levels.

**Table 6:** Linear mixed model to estimate GDF15 values.

	Univariable model		Multivariable model	
	Estimate	95% CI	Estimate	95% CI
Baseline model				
Initial value	745.7	(678.6–819.5)	494	(426–573.3)
Age model				
Initial value: <50 years	619	(552.7–693.1)	– –	
Δ initial value: ≥50 years	356.1	(176.8–610.6)	79.6	(−15.3–228.6)
Sex model				
Initial value: female	648.1	(576.3–728.7)	– –	
Δ initial value: male	264.6	(99.4–504.0)	59.2	(−23.7–187.8)
Bariatric model				
Initial value: RYGB	807.2	(707.4–921.3)	– –	
Δ initial value: sleeve	−118	(−206.3–26.8)	−19.2	(−75.8–70.8)
Diabetic model				
Initial value: no	588.9	(533.9–649.6)	– –	
Δ initial value: yes	545	(341.2–832.4)	175.7	(−30.1–414.1)
HBP model				
Initial value: no	542	(477.5–615.1)	– –	
Δ initial value: yes	403.2	(227–652.9)	173.2	(50.9–360.7)
Metformin treatment model				
Initial value: no	674.1	(613.4–742)	– –	
Δ initial value: yes	318.5	(167.3–522)	81.8	(−19.4–233.5)

## Discussion

This study aimed to evaluate the changes in circulating GDF15 levels in obesity, to assess their correlations with anthropometric, clinical, and biochemical parameters, and to determinate the impact of BS on these values and associated correlations.

In this study, we demonstrated that circulating GDF15 levels increase with obesity regardless of sex, with levels consistently higher in men. These findings are in agreement with previous data showing particularly elevated GDF15 levels in obese and elderly men (Dolo *et al.* 2020, Asrih *et al.* 2022, Mattia *et al.* 2023) and a positive correlation with body weight and adipose tissue mass (Lockhart *et al.* 2020, Asrih *et al.* 2023, Li *et al.* 2024*a*). However, some studies reported conflicting findings. For instance, a recent study observed that, in contrast to men, women exhibit a negative trend regarding the correlation of circulating GDF15 levels and obesity (Asrih *et al.* 2022). Moreover, in non-obese monozygotic twins (both sexes), circulating GDF15 levels have been found to be inversely correlated with BMI (Tsai *et al.* 2015). These data seem to suggest that increased circulating levels of GDF15 in humans are associated with decreased body weight. Studies in rodents have also observed that circulating concentrations of GDF15 are higher in obese group compared to lean controls (Wang *et al.* 2021). Furthermore, cancer-associated anorexia/cachexia syndrome has been linked to high circulating concentrations of GDF15 (Johnen *et al.* 2007), while blockade of GDF15 or its receptor, GFRAL, reduces anorexia and weight loss in rodents (Wang *et al.* 2021). Therefore, the increase in circulating levels of GDF15 during obesity could be a consequence and not a cause of it. It has been proposed that, under conditions of over-nutrition, GDF15 overexpression acts as a compensatory mechanism to mitigate damage by negatively regulating energy balance (Li *et al.* 2024*a*) and that fatty acids can stimulate GDF15 production to reduce food intake by functioning as a fatty acid sensor designed to protect cells from fatty acid overload (Wang *et al.* 2024). These findings could explain the correlations observed in this study. While a positive association between GDF15 and weight, BMI, and fat mass is evident when controls and obese subjects are analysed together, the negative correlation between GDF15 and body fat percentage observed when focussing exclusively on the cohort of patients with obesity suggests that GDF15 levels may reflect compensatory mechanisms related to adiposity regulation in individuals with obesity.

Several studies have reported an increase in circulating GDF15 levels following BS, and some have suggested that weight loss induced by surgery is linked to elevated circulating GDF15 values (Vila *et al.* 2011, Kleinert *et al.* 2019, Dolo *et al.* 2020, Salman *et al.* 2021). In contrast, our study found that circulating GDF15 levels significantly decreased 1 year after BS, regardless of sex and the type of surgery performed. However, Chaiyasoot *et al.* found that 1 year after weight loss interventions, whether through pharmacological treatment (topiramate) or BS (RYGB and SG), the reduction in circulating GDF15 levels was linked to greater weight loss (Chaiyasoot *et al.* 2023). In addition, a cross-sectional study comparing individuals who underwent BS (RYGB and SG) with healthy controls found no significant changes in fasting or postprandial circulating GDF15 levels due to BS (Martinussen *et al.* 2021). In this work, 1 year after surgery, no correlations were observed between circulating GDF15 levels and the analysed anthropometric data. However, a negative correlation was noted between the variation in circulating GDF15 levels and BMI, along with a positive correlation with the % EWL and % EBMIL, suggesting that patients with smaller reductions in circulating GDF15 experienced greater % EWL and % EBMIL.

We propose that a possible explanation for these discrepancies lies in the baseline condition of the study subjects, particularly the comorbidities they experience, the duration of these conditions, and the treatments they receive. This aligns with recent observations by Di Vincenzo *et al.* who reported that SG significantly increased circulating GDF15 levels in patients with obesity but without metabolic syndrome, while patients with metabolic syndrome exhibited higher baseline GDF15 levels that did not further increase post-surgery (Di Vincenzo *et al.* 2025). Univariate mixed linear models revealed that T2D, HBP, and metformin treatment (in addition to sex and age) significantly influence circulating GDF15 levels. However, it must be noted that, in a multivariate model adjusting for other variables, only hypertension remained a significant predictor. The mixed linear models in this study showed that patients with HBP and T2D (most of whom were treated with metformin) exhibited higher circulating GDF15 levels (>1,100 pg/mL and 1,500 pg/mL, respectively) compared to those without these conditions (<700 pg/mL). In addition, 1 year after BS, only patients with pre-existing T2D or HBP demonstrated statistically significant reductions in circulating GDF15 levels. The elevated baseline levels of circulating GDF15 observed in patients with T2D and HBP could be partially attributed to its role as a stress-induced cytokine linked to inflammation (Carstensen *et al.* 2010, Reyes & Yap 2023). Chronic low-grade inflammation characterises both conditions, and the subsequent reduction in GDF15 after BS may indicate resolution of systemic inflammatory processes, along with improved metabolic status following the remission of T2D and better control of HBP. Indeed, a substantial proportion of patients in our cohort were no longer diabetic or hypertensive at 12 months post-surgery. This hypothesis is supported by previous studies reporting that lower baseline GDF15 concentrations (200–500 pg/mL) tend to increase post-surgery (Vila *et al.* 2011, Kleinert *et al.* 2019, Dolo *et al.* 2020, Salman *et al.* 2021). However, studies with higher initial values (>1,100 pg/mL) describe decreases comparable to our findings (Chaiyasoot *et al.* 2023) or no changes (Di Vincenzo *et al.* 2025). These observations suggest that GDF15 may not only serve as a metabolic biomarker but may also reflect an individual’s baseline inflammatory and cardiometabolic burden.

This is in line with previous research suggesting that GDF15 plays a protective role in inflammation and oxidative stress, such as that seen in cardiovascular and renal diseases (Asrih *et al.* 2023, Lasaad & Crambert 2024). We observed positive correlations between circulating GDF15 levels and renal markers (urea, creatinine, uric acid, and ions) in both normal-weight and obese groups before undergoing BS. Most of these correlations disappeared after BS, though some re-emerged at 6 or 12 months. We also found positive associations with glucose, HbA1c, and triglycerides, but not with HOMA-IR, HDL, or LDL cholesterol, which is consistent with prior studies (Vila *et al.* 2011, Dolo *et al.* 2020, Ho *et al.* 2023, Boutari *et al.* 2024). A negative correlation with total-, HDL-, and LDL-cholesterol and apolipoprotein A was observed only when data from normal-weight and obese patients before surgery were combined. Altogether, these patterns support the idea that GDF15 serves as a marker of systemic metabolic stress or inflammation, rather than acting as a direct regulator of glucose or lipid metabolism.

It is well-established that circulating GDF15 levels increase in humans with MASLD and metabolic dysfunction-associated steatohepatitis (MASH) (Galuppo *et al.* 2022, Valenzuela-Vallejo *et al.* 2022, Asrih *et al.* 2023, L'Homme *et al.* 2024, Li *et al.* 2024*a*,*b*). However, the specific cell types that contribute to this increase remain unclear. Studies on human liver biopsies from individuals with cirrhosis (Ramachandran *et al.* 2019) or MASH (Govaere *et al.* 2020) have shown increased GDF15 expression across almost all liver cell types. Given the close correlation between obesity and MASLD, the liver could be a contributing tissue for the elevated circulating levels of GDF15, which could explain the positive correlations observed in this study between circulating GDF15 levels and various hepatic markers, such as alkaline phosphatase and transaminases. Adolph *et al.* investigated GDF15 expression in the liver and subcutaneous adipose tissue (SAT) of obese patients before and after undergoing adjustable gastric banding and found that while hepatic expression decreased with weight loss, adipose tissue expression remained stable (Adolph *et al.* 2018). In addition, L'Homme *et al.* using mouse models of obesity and MASLD, and biopsies from patients with obesity, T2D and MASLD highlighted a dual role for WAT and liver in GDF15 production during metabolic disease status. They reported that WAT is the primary source of GDF15 at the onset of obesity and T2D (due to macrophage accumulation), while the liver becomes more involved during progression to MASH, with GDF15 expression rising in hepatocytes as the disease advances (L'Homme *et al.* 2024). In humans, circulating GDF15 levels were found to change according to the liver fat content (Galuppo *et al.* 2022) and were significantly associated with the risk of advanced fibrosis but not with the risk of MASH (Koo & Guan 2018). Using GDF15-transgenic mice, Kim *et al.* suggested that increased GDF15 acts as a compensatory mechanism to limit the progression of MASLD (Kim *et al.* 2018). All these data suggest that the duration of obesity and the liver health of the patients may influence circulating GDF15 levels. Thus, the effectiveness of BS in reducing fat mass and resolving obesity-related comorbidities (such as T2D, HBP, or fatty liver) could lead to changes in both circulating GDF15 and its primary tissue source. Depending on the source of origin, GDF15 may exert different metabolic functions, potentially explaining discrepancies among studies examining the impact of obesity and BS on circulating GDF15 levels.

Pharmacological treatments, particularly metformin, must be considered when interpreting changes in circulating GDF15 levels. Metformin has been shown to significantly increase GDF15 levels, and part of its beneficial effects on appetite, weight loss, and insulin sensitivity appear to be mediated by this cytokine (Day *et al.* 2019, Coll *et al.* 2020). In our cohort, 36% of patients had T2D, and among them, 90% were receiving metformin before surgery, which may have contributed to the elevated baseline GDF15 levels observed. Following BS, many patients discontinued metformin due to T2D remission, potentially contributing to the observed decline in GDF15 concentrations. While this reduction likely reflects improved metabolic health and decreased systemic inflammation, the withdrawal of metformin may also have played a role.

Metformin weight-loss effects have been associated with elevated circulating GDF15 levels, although the specific contributions and tissue sources of GDF15 remain debated (Wang *et al.* 2021, Bruce *et al.* 2024). In rodents, Zhang *et al.* observed that metformin raises GDF15 via kidney synthesis, which activates GFRAL in the AP, reducing food intake and weight (Zhang *et al.* 2023*a*). However, other works do not agree with this statement. Klein *et al.* reported that metformin-induced weight loss in mice was independent of the GDF15-GFRAL pathway, and that in humans with a BMI over 25, metformin elevated GDF15 without affecting weight loss (Klein *et al.* 2022). Other authors described the gastrointestinal tract as a key source of metformin-induced GDF15 (Coll *et al.* 2020, Kincaid *et al.* 2024). Coll *et al.* proposed that metformin modulates both energy intake and expenditure via GDF15, although it can still reduce glucose independently of GDF15 (Coll *et al.* 2020), While Kincaid *et al.* reported that intestinal GDF15 is not essential for limiting weight gain from a high-fat diet (Kincaid *et al.* 2024).

The strengths of the present study include its large cohort of patients with normal weight and obesity. To the best of our knowledge, this study has the largest sample size examining circulating GDF15 levels in obese patients both before and after BS. In addition, data from the two most commonly used surgeries were analysed at three different time points in both men and women. Another notable strength is the comprehensive analysis of associations between circulating GDF15 levels and a wide range of biochemical, clinical and anthropometric parameters.

This study has several limitations that should be considered when interpreting the findings. First, the obese cohort exhibited considerable heterogeneity in both comorbidities and pharmacological treatments. Although we primarily focused on T2D, HBP, and metformin use before BS, other medications may have influenced circulating GDF15 levels. Second, lifestyle-related variables such as dietary intake and physical activity were not assessed. These factors, known to modulate metabolic stress and systemic inflammation, could independently impact GDF15 levels. Notably, GDF15 has been described as an exerkine (Laurens *et al.* 2020, Labour *et al.* 2024), suggesting that differences in exercise habits could partially contribute to the observed variability. Third, the cellular and tissue sources of circulating GDF15 before and after BS remain undefined, limiting mechanistic insight. Finally, although our focus was on circulating GDF15 concentrations, it is important to acknowledge that GDF15 signals via the central receptor GFRAL. BS could influence GFRAL expression or sensitivity, thereby modulating the appetite-suppressing effects of GDF15 and contributing to interindividual variability in weight loss responses. However, data on central GFRAL dynamics post-BS are currently lacking. Future studies should aim to address these gaps.

In conclusion, we observed a significant reduction in circulating GDF15 levels following BS, particularly in patients with HBP or T2D. While this reduction may reflect the metabolic improvements associated with surgical weight loss, the withdrawal of pharmacological treatments, such as metformin, could also contribute. These findings suggest that GDF15 may be a relevant biomarker of clinical outcomes after BS. Elevated circulating GDF15 levels may also confer some protection against fat accumulation in individuals with obesity. Moreover, after BS, ΔGDF15 correlated negatively with BMI and positively with % EWL and % EBMI. Nonetheless, further research is required to clarify the mechanistic role of GDF15.

## Supplementary materials



## Declaration of interest

The authors declare that there is no conflict of interest that could be perceived as prejudicing the impartiality of the work reported.

## Funding

This work was funded by project No. PI16/00884, awarded to SS-A and FC, integrated into the National Plan for Scientific Research, Development and Technological Innovation 2013–2016 and funded by the ISCIII (Instituto de Salud Carlos III) – General Sub-Direction of the Assessment and Promotion of the Research – European Regional Development Fund (FEDER), ‘A way of making Europe’. The Galician Public Foundation for Biomedical Research INIBIC has provided funding for the publication of the article.

## Author contribution statement

SS-A conceived the study. SS-A, VB-B, PJ-V, FC and PU designed the methodology. SS-A, VB-B, PJ-V and PU perfomed formal analysis. SS-A, PJ-V, PU, EO-B and MJG-B were involved in the investigation. SS-A, EO-B and MJG-B helped in acquiring resources. SS-A wrote the original draft of the manuscript. SS-A reviewed and edited the manuscript. SS-A, PU, VB-B and PJ-V were involved in visualization. SS-A supervised the study. SS-A and FC acquired funding. All authors have read and agreed to the published version of the manuscript.

## Ethical approval

All procedures performed in studies involving human participants were in accordance with the ethical standards of the institutional and/or national research committee and with the 1964 Declaration of Helsinki.

## References

[bib1] Adolph TE, Grabherr F, Mayr L, et al. 2018 Weight loss induced by bariatric surgery restricts hepatic GDF15 expression. J Obes 2018 7108075. (10.1155/2018/7108075) 30533221 PMC6250003

[bib2] Apovian CM 2016 Obesity: definition, comorbidities, causes, and burden. Am J Manag Care 22 s176–s185. 27356115

[bib3] Asrih M, Sinturel F, Dubos R, et al. 2022 Sex-specific modulation of circulating growth differentiation factor-15 in patients with type 2 diabetes and/or obesity. Endocr Connect 11 e220054. (10.1530/ec-22-0054) 35700236 PMC9346339

[bib4] Asrih M, Wei S, Nguyen TT, et al. 2023 Overview of growth differentiation factor 15 in metabolic syndrome. J Cell Mol Med 27 1157–1167. (10.1111/jcmm.17725) 36992609 PMC10148061

[bib5] Boutari C, Stefanakis K, Simati S, et al. 2024 Circulating total and H-specific GDF15 levels are elevated in subjects with MASLD but not in hyperlipidemic but otherwise metabolically healthy subjects with obesity. Cardiovasc Diabetol 23 174. (10.1186/s12933-024-02264-5) 38762719 PMC11102634

[bib6] Bruce K, Garrido AN, Zhang SY, et al. 2024 Regulation of energy and glucose homeostasis by the nucleus of the solitary tract and the area postrema. Endocrinol Metab 39 559–568. (10.3803/enm.2024.2025) PMC11377841 39086274

[bib7] Carstensen M, Herder C, Brunner EJ, et al. 2010 Macrophage inhibitory cytokine-1 is increased in individuals before type 2 diabetes diagnosis but is not an independent predictor of type 2 diabetes: the Whitehall II study. Eur J Endocrinol 162 913–917. (10.1530/eje-09-1066) 20167682

[bib8] Cercato C & Fonseca FA 2019 Cardiovascular risk and obesity. Diabetol Metab Syndr 11 74. (10.1186/s13098-019-0468-0) 31467596 PMC6712750

[bib9] Chaiyasoot K, Khumkhana N, Deekum W, et al. 2023 Alteration of BDNF, SPARC, FGF-21, and GDF-15 circulating levels after 1 year of anti-obesity treatments and their association with 1-year weight loss. Endocrine 82 57–68. (10.1007/s12020-023-03435-2) 37436597 PMC10462550

[bib10] Chrysovergis K, Wang X, Kosak J, et al. 2014 NAG-1/GDF-15 prevents obesity by increasing thermogenesis, lipolysis and oxidative metabolism. Int J Obes 38 1555–1564. (10.1038/ijo.2014.27) PMC4135041 24531647

[bib11] Coll AP, Chen M, Taskar P, et al. 2020 GDF15 mediates the effects of metformin on body weight and energy balance. Nature 578 444–448. (10.1038/s41586-019-1911-y) 31875646 PMC7234839

[bib12] Day EA, Ford RJ, Smith BK, et al. 2019 Metformin-induced increases in GDF15 are important for suppressing appetite and promoting weight loss. Nat Metab 1 1202–1208. (10.1038/s42255-019-0146-4) 32694673

[bib13] Di Vincenzo A, Granzotto M, Trevellin E, et al. 2025 Sleeve gastrectomy preferentially increases GDF15 plasma levels in patients with obesity but without metabolic syndrome. Obes Surg 35 341–344. (10.1007/s11695-024-07625-3) 39661245

[bib14] Dolo PR, Yao L, Liu PP, et al. 2020 Effect of sleeve gastrectomy on plasma growth differentiation factor-15 (GDF15) in human. Am J Surg 220 725–730. (10.1016/j.amjsurg.2020.01.041) 32014297

[bib15] Dong XC & Xu DY 2024 Research progress on the role and mechanism of GDF15 in body weight regulation. Obes Facts 17 1–11. (10.1159/000535089) 37989122 PMC10836939

[bib16] Emmerson PJ, Wang F, Du Y, et al. 2017 The metabolic effects of GDF15 are mediated by the orphan receptor GFRAL. Nat Med 23 1215–1219. (10.1038/nm.4393) 28846098

[bib17] Frikke-Schmidt H, Hultman K, Galaske JW, et al. 2019 GDF15 acts synergistically with liraglutide but is not necessary for the weight loss induced by bariatric surgery in mice. Mol Metab 21 13–21. (10.1016/j.molmet.2019.01.003) 30685336 PMC6407365

[bib18] Galuppo B, Agazzi C, Pierpont B, et al. 2022 Growth differentiation factor 15 (GDF15) is associated with non-alcoholic fatty liver disease (NAFLD) in youth with overweight or obesity. Nutr Diabetes 12 9. (10.1038/s41387-022-00187-2) 35194014 PMC8863897

[bib19] Ghidewon M, Wald HS, McKnight AD, et al. 2022 Growth differentiation factor 15 (GDF15) and semaglutide inhibit food intake and body weight through largely distinct, additive mechanisms. Diabetes Obes Metab 24 1010–1020. (10.1111/dom.14663) 35129264 PMC9796095

[bib20] Govaere O, Cockell S, Tiniakos D, et al. 2020 Transcriptomic profiling across the nonalcoholic fatty liver disease spectrum reveals gene signatures for steatohepatitis and fibrosis. Sci Transl Med 12 eaba4448. (10.1126/scitranslmed.aba4448) 33268509

[bib21] Ho LC, Wu HT, Hung HC, et al. 2023 Growth differentiation factor-15 is independently associated with metabolic syndrome and hyperglycemia in non-elderly subjects. Biofactors 49 119–126. (10.1002/biof.1871) 35686301

[bib22] Hsu JY, Crawley S, Chen M, et al. 2017 Non-homeostatic body weight regulation through a brainstem-restricted receptor for GDF15. Nature 550 255–259. (10.1038/nature24042) 28953886

[bib23] Johnen H, Lin S, Kuffner T, et al. 2007 Tumor-induced anorexia and weight loss are mediated by the TGF-beta superfamily cytokine MIC-1. Nat Med 13 1333–1340. (10.1038/nm1677) 17982462

[bib24] Kim KH, Kim SH, Han DH, et al. 2018 Growth differentiation factor 15 ameliorates nonalcoholic steatohepatitis and related metabolic disorders in mice. Sci Rep 8 6789. (10.1038/s41598-018-25098-0) 29717162 PMC5931608

[bib25] Kincaid JWR, Rimmington D, Tadross JA, et al. 2024 The gastrointestinal tract is a major source of the acute metformin-stimulated rise in GDF15. Sci Rep 14 1899. (10.1038/s41598-024-51866-2) 38253650 PMC10803367

[bib26] Klein AB, Nicolaisen TS, Johann K, et al. 2022 The GDF15-GFRAL pathway is dispensable for the effects of metformin on energy balance. Cell Rep 40 111258. (10.1016/j.celrep.2022.111258) 36001956

[bib27] Kleinert M, Bojsen-Moller KN, Jorgensen NB, et al. 2019 Effect of bariatric surgery on plasma GDF15 in humans. Am J Physiol Endocrinol Metab 316 E615–E621. (10.1152/ajpendo.00010.2019) 30721097

[bib28] Koo JH & Guan KL 2018 Interplay between YAP/TAZ and metabolism. Cell Metab 28 196–206. (10.1016/j.cmet.2018.07.010) 30089241

[bib29] L'Homme L, Sermikli BP, Haas JT, et al. 2024 Adipose tissue macrophage infiltration and hepatocyte stress increase GDF-15 throughout development of obesity to MASH. Nat Commun 15 7173. (10.1038/s41467-024-51078-2) 39169003 PMC11339436

[bib30] Labour A, Lac M, Frassin L, et al. 2024 GDF15 is dispensable for the insulin-sensitizing effects of chronic exercise. Cell Rep 43 114577. (10.1016/j.celrep.2024.114577) 39096490

[bib31] Lasaad S & Crambert G 2024 GDF15, an emerging player in renal physiology and pathophysiology. Int J Mol Sci 25 5956. (10.3390/ijms25115956) 38892145 PMC11172470

[bib32] Laurens C, Parmar A, Murphy E, et al. 2020 Growth and differentiation factor 15 is secreted by skeletal muscle during exercise and promotes lipolysis in humans. JCI Insight 5 e131870. (10.1172/jci.insight.131870) 32106110 PMC7213799

[bib33] Li J, Hu X, Xie Z, et al. 2024a Overview of growth differentiation factor 15 (GDF15) in metabolic diseases. Biomed Pharmacother 176 116809. (10.1016/j.biopha.2024.116809) 38810400

[bib34] Li Y, Zhang J, Chen S, et al. 2024b Growth differentiation factor 15: emerging role in liver diseases. Cytokine 182 156727. (10.1016/j.cyto.2024.156727) 39111112

[bib35] Lockhart SM, Saudek V & O'Rahilly S 2020 GDF15: a hormone conveying somatic distress to the brain. Endocr Rev 41 bnaa007. (10.1210/endrev/bnaa007) 32310257 PMC7299427

[bib36] Macia L, Tsai VW, Nguyen AD, et al. 2012 Macrophage inhibitory cytokine 1 (MIC-1/GDF15) decreases food intake, body weight and improves glucose tolerance in mice on normal & obesogenic diets. PLoS One 7 e34868. (10.1371/journal.pone.0034868) 22514681 PMC3325923

[bib37] Martinussen C, Svane MS, Bojsen-Moller KN, et al. 2021 Plasma GDF15 levels are similar between subjects after bariatric surgery and matched controls and are unaffected by meals. Am J Physiol Endocrinol Metab 321 E443–E452. (10.1152/ajpendo.00190.2021) 34370594

[bib38] Mattia L, Gossiel F, Walsh JS, et al. 2023 Effect of age and gender on serum growth differentiation factor 15 and its relationship to bone density and bone turnover. Bone Rep 18 101676. (10.1016/j.bonr.2023.101676) 37090856 PMC10113774

[bib39] Mullican SE, Lin-Schmidt X, Chin CN, et al. 2017 GFRAL is the receptor for GDF15 and the ligand promotes weight loss in mice and nonhuman primates. Nat Med 23 1150–1157. (10.1038/nm.4392) 28846097

[bib40] Ramachandran P, Dobie R, Wilson-Kanamori JR, et al. 2019 Resolving the fibrotic niche of human liver cirrhosis at single-cell level. Nature 575 512–518. (10.1038/s41586-019-1631-3) 31597160 PMC6876711

[bib41] Reyes J & Yap GS 2023 Emerging roles of growth differentiation factor 15 in immunoregulation and pathogenesis. J Immunol 210 5–11. (10.4049/jimmunol.2200641) 36542831 PMC9779231

[bib42] Salman A, Shaaban HE, Salman M, et al. 2021 Changes in plasma growth differentiation factor-15 after laparoscopic sleeve gastrectomy in morbidly obese patients: a prospective study. J Inflamm Res 14 1365–1373. (10.2147/jir.s304929) 33880052 PMC8052116

[bib43] Sjoberg KA, Sigvardsen CM, Alvarado-Diaz A, et al. 2023 GDF15 increases insulin action in the liver and adipose tissue via a beta-adrenergic receptor-mediated mechanism. Cell Metab 35 1327–1340.e5. (10.1016/j.cmet.2023.06.016) 37473755

[bib44] Suriben R, Chen M, Higbee J, et al. 2020 Antibody-mediated inhibition of GDF15-GFRAL activity reverses cancer cachexia in mice. Nat Med 26 1264–1270. (10.1038/s41591-020-0945-x) 32661391

[bib45] Tran T, Yang J, Gardner J, et al. 2018 GDF15 deficiency promotes high fat diet-induced obesity in mice. PLoS One 13 e0201584. (10.1371/journal.pone.0201584) 30070999 PMC6072047

[bib46] Tsai VW, Macia L, Johnen H, et al. 2013 TGF-b superfamily cytokine MIC-1/GDF15 is a physiological appetite and body weight regulator. PLoS One 8 e55174. (10.1371/journal.pone.0055174) 23468844 PMC3585300

[bib47] Tsai VW, Macia L, Feinle-Bisset C, et al. 2015 Serum levels of human MIC-1/GDF15 vary in a diurnal pattern, do not display a profile suggestive of a satiety factor and are related to BMI. PLoS One 10 e0133362. (10.1371/journal.pone.0133362) 26207898 PMC4514813

[bib48] Tsai VW, Zhang HP, Manandhar R, et al. 2018 Treatment with the TGF-b superfamily cytokine MIC-1/GDF15 reduces the adiposity and corrects the metabolic dysfunction of mice with diet-induced obesity. Int J Obes 42 561–571. (10.1038/ijo.2017.258) 29026214

[bib49] Valenzuela-Vallejo L, Chrysafi P & Mantzoros CS 2022 Liraglutide-induced effects on energy intake and glycemic profile are independent of total and intact GDF-15 levels in subjects with obesity and diabetes. Diabetes Metab 48 101369. (10.1016/j.diabet.2022.101369) 35777691

[bib50] Vila G, Riedl M, Anderwald C, et al. 2011 The relationship between insulin resistance and the cardiovascular biomarker growth differentiation factor-15 in obese patients. Clin Chem 57 309–316. (10.1373/clinchem.2010.153726) 21164037

[bib51] Wang D, Day EA, Townsend LK, et al. 2021 GDF15: emerging biology and therapeutic applications for obesity and cardiometabolic disease. Nat Rev Endocrinol 17 592–607. (10.1038/s41574-021-00529-7) 34381196

[bib52] Wang D, Townsend LK, DesOrmeaux GJ, et al. 2023 GDF15 promotes weight loss by enhancing energy expenditure in muscle. Nature 619 143–150. (10.1038/s41586-023-06249-4) 37380764 PMC10322716

[bib53] Wang D, Jabile MJT, Lu J, et al. 2024 Fatty acids increase GDF15 and reduce food intake through a GFRAL signaling axis. Diabetes 73 51–56. (10.2337/db23-0495) 37847913 PMC10784653

[bib54] Xiong Y, Walker K, Min X, et al. 2017 Long-acting MIC-1/GDF15 molecules to treat obesity: evidence from mice to monkeys. Sci Transl Med 9 eaan8732. (10.1126/scitranslmed.aan8732) 29046435

[bib55] Yang L, Chang CC, Sun Z, et al. 2017 GFRAL is the receptor for GDF15 and is required for the anti-obesity effects of the ligand. Nat Med 23 1158–1166. (10.1038/nm.4394) 28846099

[bib56] Yang Y, Miao C, Wang Y, et al. 2024 The long-term effect of bariatric/metabolic surgery versus pharmacologic therapy in type 2 diabetes mellitus patients: a systematic review and meta-analysis. Diabetes Metab Res Rev 40 e3830. (10.1002/dmrr.3830) 38873748

[bib57] Zhang SY, Bruce K, Danaei Z, et al. 2023a Metformin triggers a kidney GDF15-dependent area postrema axis to regulate food intake and body weight. Cell Metab 35 875–886.e5. (10.1016/j.cmet.2023.03.014) 37060902 PMC12272050

[bib58] Zhang Y, Zhao X, Dong X, et al. 2023b Activity-balanced GLP-1/GDF15 dual agonist reduces body weight and metabolic disorder in mice and non-human primates. Cell Metab 35 287–298.e4. (10.1016/j.cmet.2023.01.001) 36706758

