# Supplementary material for: Relevance of GDF15 as a biomarker for clinical outcomes after bariatric surgery
Source: J Endocrinol. 2025 Jul 1;266(1):e250010. doi: 10.1530/JOE-25-0010 (PMC12231176; doi:10.1530/JOE-25-0010)
Supplement: Supplementary file 1 [file supplementary_materials.pdf]

Supplementary Table 1. Spearman correlations between circulating GDF15 values and different biochemical parameters. Both the global correlations (control patients + patients with obesity before bariatric surgery) and the correlations in patients with obesity before and after bariatric surgery are shown.

	**Global**				**Before bariatric surgery**				**3 months after bariatric surgery**				**6 months after bariatric surgery**				**12 months after bariatric surgery**			
	**Rho Spearman**	**p**	**95% IC**		**Rho Spearman**	**p**	**95% IC**		**Rho Spearman**	**p**	**95% IC**		**Rho Spearman**	**p**	**95% IC**		**Rho Spearman**	**p**	**95% IC**	
GDF15 - Glucose	**0.270**	**<.001**	0.127	0.402	**0.384**	**<.001**	0.219	0.528	**0.362**	**0.003**	0.120	0.563	0.221	0.068	-0.023	0.441	**0.255**	**0.010**	0.058	0.433
GDF15 - Insulin	**0.326**	**<.001**	0.180	0.458	**0.329**	**<.001**	0.153	0.485	0.071	0.582	-0.187	0.320	0.084	0.497	-0.166	0.325	0.027	0.788	-0.175	0.227
GDF15 - C-peptide	**0.550**	**<.001**	0.432	0.650	**0.484**	**<.001**	0.328	0.614	0.182	0.153	-0.076	0.418	0.161	0.192	-0.089	0.393	**0.220**	**0.027**	0.020	0.403
GDF15 - HbA1c (Glycated haemoglobin)	**0.551**	**<.001**	0.414	0.665	**0.473**	**<.001**	0.297	0.617	**0.304**	**0.028**	0.026	0.539	**0.288**	**0.028**	0.024	0.514	**0.273**	**0.011**	0.060	0.462
GDF15 - HOMA-IR	**0.320**	**<.001**	0.174	0.453	**0.341**	**<.001**	0.166	0.495	0.145	0.253	-0.112	0.384	0.104	0.403	-0.147	0.342	0.081	0.418	-0.122	0.278
GDF15 - Cholesterol	**-0.313**	**<.001**	-0.445	-0.168	0.007	0.936	-0.176	0.190	-0.012	0.925	-0.264	0.241	-0.163	0.181	-0.391	0.084	0.064	0.520	-0.137	0.261
GDF15 - HDL-Cholesterol	**-0.495**	**<.001**	-0.604	-0.369	-0.136	0.139	-0.313	0.050	-0.091	0.479	-0.338	0.168	-0.163	0.185	-0.392	0.086	-0.190	0.059	-0.379	0.013
GDF15 - LDL-Cholesterol	**-0.275**	**<.001**	-0.413	-0.126	-0.086	0.350	-0.266	0.100	-0.017	0.896	-0.277	0.245	-0.166	0.183	-0.398	0.087	0.101	0.318	-0.104	0.298
GDF15 - Triglycerides	**0.477**	**<.001**	0.349	0.587	**0.290**	**0.001**	0.113	0.449	**0.366**	**0.003**	0.125	0.566	**0.294**	**0.014**	0.054	0.501	**0.306**	**0.002**	0.113	0.477
GDF15 - Apolipoprotein A	**-0.359**	**<.001**	-0.487	-0.217	0.114	0.214	-0.072	0.292	-0.011	0.937	-0.283	0.263	**-0.270**	**0.034**	-0.493	-0.014	-0.160	0.120	-0.354	0.048
GDF15 - Apolipoprotein B	-0.026	0.735	0.180	0.129	0.163	0.074	-0.022	0.338	0.094	0.493	-0.183	0.358	-0.169	0.188	-0.408	0.091	0.058	0.576	-0.150	0.261
GDF15 - C-reactive protein	**0.369**	**<.001**	0.226	0.496	0.067	0.474	-0.121	0.250	-0.094	0.496	-0.357	0.184	-0.031	0.810	-0.286	0.228	0.100	0.336	-0.110	0.303
GDF15 - Urea	**0.297**	**<.001**	0.023	0.321	**0.470**	**<.001**	0.315	0.601	**0.261**	**0.037**	0.009	0.482	0.101	0.411	-0.146	0.336	**0.354**	**<.001**	0.165	0.517
GDF15 - Creatinine	**0.177**	**0.020**	0.146	0.423	**0.492**	**<.001**	0.340	0.618	0.135	0.288	-0.122	0.375	0.206	0.089	-0.039	0.428	**0.197**	**0.047**	-0.003	0.382
GDF15 - Uric acid	**0.471**	**<.001**	0.341	0.582	**0.340**	**<.001**	0.168	0.492	0.202	0.109	-0.053	0.433	0.167	0.177	-0.084	0.398	**0.213**	**0.031**	0.014	0.397
GDF15 - Albumin	-0.140	0.094	-0.301	0.029	**0.269**	**0.004**	0.085	0.434	0.010	0.939	-0.254	0.273	-0.098	0.434	-0.339	0.155	-0.078	0.445	-0.279	0.129
GDF15 - Calcium	**-0.202**	**0.008**	-0.369	-0.068	**0.302**	**0.001**	0.121	0.464	0.016	0.901	-0.244	0.274	0.142	0.254	-0.111	0.378	**0.230**	**0.02**	0.031	0.411
GDF15 - Phosphorus	-0.039	0.620	-0.196	0.119	**0.220**	**0.019**	0.032	0.393	-0.009	0.943	-0.268	0.250	0.128	0.305	-0.125	0.365	0.190	0.056	-0.011	0.375
GDF15 - Sodium	**-0.202**	**0.008**	-0.345	-0.049	-0.124	0.175	-0.300	0.061	0.183	0.147	-0.073	0.417	0.054	0.659	-0.192	0.294	0.185	0.063	-0.016	0.371
GDF15 - Potassium	**0.220**	**0.004**	0.067	0.362	0.048	0.600	-0.137	0.230	-0.036	0.779	-0.291	0.222	0.070	0.568	-0.178	0.310	0.058	0.565	-0.144	0.255
GDF15 - Chlorine	**-0.235**	**0.002**	-0.375	-0.084	**-0.333**	**<.001**	-0.486	-0.160	-0.129	0.310	-0.370	0.128	-0.089	0.469	-0.327	0.159	0.010	0.919	-0.191	0.211
GDF15 – AP (Alkaline phosphatase)	**0.147**	**0.089**	-0.028	0.314	**0.182**	**0.048**	-0.004	0.356	0.055	0.666	-0.200	0.304	**0.278**	**0.022**	0.035	0.490	0.038	0.708	-0.165	0.238
GDF15 - AST (Aspartate aminotransferase)	**0.557**	**<.001**	0.439	0.656	**0.408**	**<.001**	0.242	0.551	0.218	0.089	-0.041	0.450	**0.341**	**0.004**	0.105	0.541	0.121	0.231	-0.083	0.315
GDF15 - ALT (Alanine aminotransferase)	**0.486**	**<.001**	0.357	0.596	**0.329**	**<.001**	0.156	0.483	0.084	0.509	-0.172	0.330	**0.302**	**0.012**	0.062	0.510	0.050	0.618	-0.153	0.250
GDF15 - GGT (Gamma-	**0.339**	**<.001**	0.187	0.476	**0.252**	**0.005**	0.073	0.416	0.237	0.059	-0.016	0.462	**0.267**	**0.027**	0.024	0.481	0.076	0.454	-0.128	0.274

glutamyl transferase)																				
GDF15 - Total bilirubin	0.033	0.698	-0.139	0.204	0.113	0.235	-0.079	0.296	-0.077	0.575	-0.343	0.200	-0.147	0.255	-0.389	0.114	-0.122	0.233	-0.319	0.085
GDF15 - Iron	-0.114	0.245	-0.303	0.084	0.082	0.441	-0.133	0.290	-0.017	0.897	-0.270	0.239	0.013	0.919	-0.233	0.257	-0.186	0.066	-0.376	0.018
GDF15 - TIBC (Total iron binding capacity)	0.123	0.203	-0.072	0.310	**0.273**	**0.008**	0.066	0.458	0.053	0.677	-0.204	0.304	0.007	0.953	-0.238	0.252	0.003	0.977	-0.201	0.207
GDF15 - Iron saturation	-0.119	0.226	-0.308	0.080	0.007	0.951	-0.207	0.219	-0.035	0.787	-0.287	0.222	0.008	0.950	-0.238	0.252	-0.189	0.064	-0.379	0.017
GDF15 - Ferritin	**0.207**	**0.032**	0.012	0.387	0.146	0.168	-0.068	0.347	0.006	0.963	-0.249	0.260	-0.034	0.784	-0.277	0.213	0.055	0.588	-0.150	0.257
GDF15 - GH (Growth hormone)	-0.026	0.736	-0.180	0.129	-0.004	0.967	-0.189	0.181	0.012	0.925	-0.245	0.268	0.011	0.929	-0.242	0.263	-0.011	0.914	-0.214	0.193
GDF15 - IGF1 (Insulin-like growth factor 1)	**-0.373**	**<.001**	-0.500	-0.231	-0.043	0.640	-0.228	0.144	**-0.265**	**0.037**	-0.489	-0.009	**-0.391**	**0.001**	-0.583	-0.158	-0.145	0.155	-0.339	0.061
GDF15 - free T4 (Tiroxin)	**0.441**	**<.001**	0.308	0.558	**0.183**	**0.042**	0.001	0.354	0.050	0.716	-0.226	0.319	-0.081	0.526	-0.327	0.176	0.103	0.316	-0.105	0.302
GDF15 - TSH (Thyroid stimulating hormone)	-0.007	0.925	-0.160	0.146	0.028	0.757	-0.155	0.209	-0.110	0.416	-0.367	0.163	0.028	0.825	-0.224	0.277	0.127	0.215	-0.080	0.324
GDF15 - Cortisol	0.029	0.704	-0.126	0.183	0.128	0.162	-0.057	0.306	-0.022	0.871	-0.293	0.252	0.046	0.719	-0.211	0.298	**0.227**	**0.027**	0.021	0.415
GDF15 - Testosterone	0.042	0.592	-0.116	0.198	**0.202**	**0.030**	0.014	0.376	0.091	0.514	-0.189	0.357	0.105	0.412	-0.154	0.351	0.137	0.189	-0.074	0.336
GDF15 - Estradiol	-0.105	0.228	-0.274	0.071	0.004	0.968	-0.210	0.218	**-0.371**	**0.006**	-0.589	-0.104	-0.214	0.095	-0.446	0.045	**-0.245**	**0.019**	-0.433	-0.036
GDF15 - Testosterone/Estradiol	0.121	0.166	-0.055	0.290	**0.237**	**0.026**	0.023	0.431	**0.352**	**0.010**	0.082	0.574	0.213	0.097	-0.047	0.445	**0.301**	**0.004**	0.096	0.481
GDF15 - 25-Hydroxyvitamin D	-0.037	0.675	-0.213	0.141	0.023	0.815	-0.177	0.221	-0.051	0.691	-0.300	0.205	0.007	0.956	-0.239	0.252	-0.020	0.845	-0.219	0.181
GDF15 - Folic acid	**0.178**	**0.039**	0.004	0.340	0.144	0.138	-0.052	0.329	**0.288**	**0.022**	0.036	0.506	0.050	0.687	-0.198	0.291	-0.060	0.549	-0.257	0.142
GDF15 - Vitamin B12	-0.085	0.321	0.254	0.089	-0.039	0.687	-0.231	0.156	-0.098	0.443	-0.342	0.159	-0.056	0.651	-0.297	0.192	-0.099	0.323	-0.295	0.104
GDF15 - Leukocytes	0.114	0.163	-0.051	0.273	0.142	0.121	-0.043	0.317	0.128	0.313	-0.129	0.369	0.130	0.291	-0.119	0.364	0.146	0.142	-0.055	0.335
GDF15 - Erythrocytes	0.076	0.353	-0.089	0.237	0.161	0.078	-0.023	0.335	-0.061	0.631	-0.309	0.195	0.022	0.861	-0.225	0.265	0.004	0.969	-0.195	0.203
GDF15 - Hemoglobin	0.042	0.606	-0.123	0.205	0.178	0.051	-0.006	0.350	0.028	0.824	-0.226	0.279	0.190	0.121	-0.058	0.416	0.077	0.439	-0.124	0.272
GDF15 - Hematocrit	0.023	0.775	-0.141	0.187	0.163	0.074	-0.022	0.336	-0.004	0.975	-0.257	0.249	0.081	0.509	-0.167	0.320	0.092	0.354	-0.109	0.286
GDF15 - MCV (Mean corpuscular volume)	**-0.164**	**0.044**	-0.319	-0.000	-0.067	0.464	-0.248	0.118	0.076	0.552	-0.18	0.322	0.001	0.993	-0.244	0.246	0.129	0.197	-0.073	0.321
GDF15 - MCH (Mean corpuscular hemoglobin)	-0.118	0.148	-0.276	0.047	-0.043	0.640	-0.225	0.142	0.069	0.587	-0.187	0.316	0.116	0.346	-0.133	0.351	0.092	0.360	-0.110	0.286
GDF15 - MCHC (Mean corpuscular hemoglobin concentration)	0.039	0.630	-0.125	0.202	0.028	0.757	-0.156	0.211	0.023	0.859	-0.231	0.274	0.189	0.123	-0.059	0.415	-0.030	0.764	-0.229	0.171
GDF15 - Platelets	-0.026	0.747	-0.189	0.138	-0.041	0.658	-0.223	0.144	0.023	0.855	-0.231	0.274	-0.020	0.874	-0.263	0.227	-0.022	0.828	-0.221	0.179
GDF15 - MPV (Mean platelet volume)	**0.211**	**0.009**	0.049	0.363	**0.206**	**0.023**	0.023	0.375	-0.113	0.376	-0.358	0.146	0.082	0.509	-0.167	0.320	0.027	0.786	-0.175	0.227
GDF15 - % Lymphocytes	-0.130	0.110	-0.288	0.034	-0.015	0.867	-0.198	0.169	0.056	0.660	-0.200	0.305	0.107	0.385	-0.142	0.343	-0.102	0.308	-0.296	0.100
GDF15 - % Monocytes	**-0.172**	**0.034**	-0.327	-0.009	-0.074	0.422	-0.254	0.112	0.102	0.422	-0.155	0.346	0.219	0.073	-0.028	0.441	0.150	0.133	-0.052	0.340
GDF15 - % Neutrophils	0.141	0.082	-0.023	0.298	0.026	0.779	-0.159	0.208	-0.105	0.407	-0.349	0.151	-0.140	0.256	-0.372	0.109	0.066	0.508	-0.136	0.263
GDF15 - % Eosinophils	-0.116	0.154	-0.275	0.048	-0.100	0.276	-0.278	0.085	0.149	0.240	-0.108	0.387	0.104	0.397	-0.145	0.341	0.094	0.349	-0.108	0.288
GDF15 - % Basophils	-0.055	0.500	-0.217	0.109	0.034	0.711	-0.151	0.216	**0.300**	**0.016**	0.051	0.513	-0.135	0.271	-0.368	0.114	0.005	0.962	-0.196	0.205

GDF15 - LUC/LYC (Large unstained cells over lymphocyte count)	**-0.159**	**0.04**	-0.313	0.002	-0.130	0.156	-0.306	0.055	-0.025	0.846	-0.278	0.231	**0.244**	**0.045**	-0.001	0.462	-0.057	0.570	-0.255	0.145
GDF15 - Lymphocytes	-0.023	0.775	-0.184	0.139	0.103	0.259	-0.082	0.282	0.110	0.389	-0.147	0.353	0.204	0.095	-0.044	0.428	0.099	0.321	-0.103	0.293
GDF15 - Monocytes	-0.083	0.301	-0.241	0.079	0.018	0.847	-0.166	0.201	0.225	0.074	-0.029	0.452	**0.256**	**0.035**	0.011	0.471	**0.248**	**0.012**	0.051	0.427
GDF15 - Neutrophils	**0.161**	**0.045**	-0.000	0.315	0.110	0.231	-0.076	0.287	0.085	0.503	-0.171	0.331	0.007	0.956	-0.239	0.252	0.131	0.188	-0.070	0.323
GDF15 - Eosinophils	-0.155	0.053	-0.309	0.007	-0.050	0.589	-0.231	0.135	0.213	0.092	-0.043	0.442	0.158	0.197	-0.090	0.388	0.175	0.079	-0.026	0.362
GDF15 - Basophils	-0.016	0.841	-0.177	0.146	0.116	0.204	-0.069	0.294	**0.321**	**0.010**	0.075	0.531	-0.037	0.762	-0.280	0.210	0.113	0.260	-0.089	0.306

CI: confidence intervals

Supplementary Table 2. Evolution of circulating GDF15 concentration in patients with obesity depending on the type of bariatric surgery

		Bariatric surgery								
		SG			RYGB					
		**Mean**	**SD**	**N**	**Mean**	**SD**	**N**	**p1**	**SG-p2**	**RYGB-p2**
GDF15 t0 (Total)		876.42	673.21	72	999.68	729.13	72	0.110		
GDF15 t3 (Total)		675.88	400.01	33	787.52	306.07	31	**0.034**		
GDF15 t6 (Total)		612.79	237.51	35	803.25	356.18	36	**0.009**		
GDF15 t12 (Total)		642.13	455.52	51	664.45	361.08	55	0.182		
ΔGDF15 (T3-T0) (Total)		-178.69	566.03	33	-184.54	672.12	31	0.825		
ΔGDF15 (T6-T0) (Total)		-87.73	500.58	31	-211.65	792.83	31	0.617		
ΔGDF15 (T12-T0) (Total)		-220.56	539.46	51	-278.01	645.36	55	0.735		
GDF15 t0	Men	1180.68	788.57	33	1079.55	690.69	27	0.749	**<.001**	0.146
	Women	618.97	417.99	39	951.76	754.78	45	**0.016**		
GDF15 t3	Men	858.3	590.61	11	861.09	336.65	9	0.425	0.127	0.384
	Women	584.67	227.09	22	757.43	295.61	22	**0.031**		
GDF15 t6	Men	735.81	343.77	8	989.14	449.99	10	0.11	0.455	0.056
	Women	576.34	189.5	27	731.75	292.53	26	**0.044**		
GDF15 t12	Men	815.56	597.61	24	784.03	538.78	19	0.922	**0.017**	0.633
	Women	487.97	175.09	27	601.33	200.01	36	**0.022**		
ΔGDF15 (T3-T0)	Men	-342.93	730.73	11	-222.9	648.55	9	0.849	0.516	0.258
	Women	-96.57	461.24	22	-168.85	695.86	22	0.981		
ΔGDF15 (T6-T0)	Men	-369.61	778.11	4	332.22	594.95	5	0.221	0.409	0.133
	Women	-45.97	453.03	27	-316.24	791.91	26	0.247		
ΔGDF15 (T12-T0)	Men	-324.37	684.63	24	-271.13	654.53	19	0.696	0.113	0.524
	Women	-128.29	355.28	27	-281.65	649.78	36	1,000		

p1: p-value for the contrast depending on the type of surgery using the Mann-Whitney U test.
P2: p-value for the contrast according to sex using the Mann-Whitney U test in each classification group according to the type of surgery.
SD, standard deviation; SG, sleeve gastrectomy; RYGB, Roux-en-Y gastric bypass.
